# The Teeth

**Published:** 1879-01

**Authors:** 


### THE TEETH.

From five to fifteen, the teeth of children should be examined by a skillful and conscientious dentist, twice a year, and thereafter at least once a year until thirty years old; by so doing, the teeth will be in a condition to last ten or fifteen years longer than they otherwise would have done, to say nothing of the advantages derived in the way of personal beauty, the promotion of good health, and the prevention and cure of dyspepsia by that thorough mastication of food, for which good teeth are essential.

Changes are constantly occurring in the practice of dentists, in consequence of the developments made as a result of scientific investigation. Tin-foil was introduced some years ago, as a cheap substitute for gold, in filling teeth, and as particularly applicable where nerves were exposed. Later on, great prejudices were excited against it, and for some years it has been discarded by the most prominent operators.

